# Investigation of the Volatile Profile of Red Jujube by Using GC-IMS, Multivariate Data Analysis, and Descriptive Sensory Analysis

**DOI:** 10.3390/foods11030421

**Published:** 2022-01-31

**Authors:** Yening Qiao, Qinqin Chen, Jinfeng Bi, Xinye Wu, Xinwen Jin, Min Gou, Xinrui Yang, Giorgia Purcaro

**Affiliations:** 1 Institute of Food Science and Technology, Chinese Academy of Agricultural Sciences (CAAS), Key Laboratory of Agro-Products Processing, Ministry of Agriculture and Rural Affairs, Beijing 100193, China; yening.qiao@student.uliege.be (Y.Q.); celerylc@163.com (Q.C.); xinye.wu@student.uliege.be (X.W.); Min.Gou@student.uliege.be (M.G.); 15003702066@163.com (X.Y.); 2 Gembloux Agro-Bio Tech Department, University of Liége, 5030 Gembloux, Belgium; 3 Institute of Agro-Products Processing Science and Technology, XinJiang Academy of Agricultural and Reclamation Science, Shihezi 832000, China; njs701022@163.com

**Keywords:** red jujube, aroma distinction, E-nose, GC-IMS, sensory attributes

## Abstract

The aroma characteristics of six red jujube cultivars (Jinchang—‘JC’, Junzao—‘JZ’, Huizao—‘HZ’, Qiyuexian—‘QYX’, Hetiandazao—‘HTDZ’, and Yuanzao—‘YZ’), cultivated in Xinjiang Province, China, were studied by E-nose and GC-IMS. The presence of acetoin, E-2-hexanol, hexanal, acetic acid, and ethyl acetate played an important role in the classification results. JC, JZ, HZ, and YZ were different from others, while QYX and HTDZ were similar to each other. HZ had the most abundant specific VOCs, including linalool, nonanoic acid, methyl myristoleate, 2-acetylfuran, 1-octen-3-one, E-2-heptenal, 2-heptenone, 7-octenoic acid, and 2-pentanone. HZ had higher intensity in jujube ID, floral, sweet, and fruity attributes. Correlation analysis showed that jujube ID (identity) might be related to phenylacetaldehyde and isobutanoic acid that formed by the transamination or dehydrogenation of amino acids; meanwhile, the sweet attribute was correlated with amino acids, including threonine, glutamic acid, glycine, alanine, valine, leucine, tyrosine, phenylalanine, lysine, histidine, and arginine.

## 1. Introduction

Jujube (*Ziziphus Jujuba* Mill.), belonging to the family Rhamnaceae, is a plant largely distributed in tropical and sub-tropical regions. In particular, it is widely distributed in China, where its fruits are regularly consumed for their good aroma, delicious taste, and high nutraceutical value in the Chinese medicine tradition. Moreover, at present, red jujubes are also widely used in the food industry (as an ingredient of tea, snacks, bread, cakes, yogurt, etc.) [1]. There are over 1000 varieties of red jujube cultivated in China, with distribution in Xinjiang, Gansu, Ningxia, Shaanxi, Shanxi, Shandong, Hebei, and Henan Provinces [2], but Xinjiang Province (latitude: 34°22′ N~49°10′ N; longitude: 73°40′ E~96°23′ E; elevation: 967.2 m~1388.78 m) is by far the largest jujube-producing region in China, with an annual output of more than 1.45 million tons in 2018 [3]. The quality of red jujubes from Xinjiang is superior to other regions because of the low rainfall, with periodic drought, abundant sunshine, and substantial differences between day and night temperatures [4]. Different cultivars of red jujubes are present in China, among which Huizao (HZ), Junzao (JZ), Yuanzao (YZ), Qiyuexian (QYX), Jinchang (JC), and Hetiandazao (HTDZ) are the most widely cultivated in different regions of Xinjiang Province. Huizao (HZ) alone accounts for 62.9% of the production from the region, followed by Junzao (JZ), which accounts for 32.7%. Moreover, Charkhlik HZ and Khotan JZ are two cultivars of protected geographical indication [3]. Several studies on phytochemical profiling, bioactive components, and pathogenic factor analysis have been carried out to better understand the quality gained by red jujube cultivars planted in Xinjiang [5,6]. Nevertheless, no studies have been conducted to investigate the particular aroma profile of the red jujube cultivated in Xinjiang Province, and to correlate the sensory characteristics with a chemical composition including VOCs, fatty acids, amino acids, organic acids, and sugars.

In fact, the primary metabolite distribution in fruits (including sugars, organic acids, and amino acids) may be associated with sensory traits such as sweetness and sourness, both as precursors of VOCs related to aroma and taste, or as involved in the browning reaction [7,8]. For example, the content of sucrose in baked potatoes had an influence on the sweetness [9]. The composition of citric, quinic, and malic acids had a strong relationship with sourness in blackcurrant juice [10]. Threonine, serine, and alanine were found to be correlated with the sweet attribute, aspartate and glutamate were correlated with the sour attribute, and valine, methionine, isoleucine, leucine, and arginine were found to contribute to the bitter taste in table grape berries [11].

E-noses have been applied to successfully discriminate the aroma qualities of food, such as the varieties of jujube and Lycium ruthenicum Murray from different provinces, harvest years, and varieties. The sum of PC1 and PC2 was in the range of 80~98% for the classifications [12,13,14]. The aim of this work is to investigate the aroma profile of red jujube cultivars cultivated in Xinjiang Province by using gas chromatography–ion mobility spectrometry (GC-IMS) and electronic noses (E-noses), and investigate the relationship between the sensory perception and the characteristic VOCs and metabolite precursors.

The data from the volatile profile was elaborated for the following reasons: (i) to discriminate among the different cultivars of red jujube (JC, JZ, HZ, QYX, HTDZ, and YZ cultivated in Xinjiang Province; (ii) to investigate the correlation between sensory attributes and chemical compositions including VOCs, fatty acids, amino acids, organic acids, and sugars.

## 2. Materials and Methods

### 2.1. Plant Material Preparation

Six cultivars of red jujubes (*Ziziphus Jujuba* Mill., JC, JZ, HZ, QYX, HTDZ, and YZ, each cultivar with three biological repeats), which were grown in the Xinjiang Province of China, were used in this study (Table S1). The botanical identification was confirmed by expert botanists. Fifty kilograms of each cultivar of red jujube were bought at mature commercial stage in the Beiyuanchun Jujube Market (Urumqi, China, 2018). Red jujubes without physical damage and/or infections were selected and stored at 4 °C before analysis within 72 h.

### 2.2. Chemicals and Regents

The chemical standards of formic acid, hexanoic acid, propionic acid, acetic acid, isobutyric acid, 3-methylbutanoic acid, pentanoic acid, 3-heptenoic acid, nonanoic acid, crotonic acid, 7-octenoic acid, n-decanoic acid, heptanoic acid, 2-heptenoic acid, ethanol, linalool, E-2-hexenol, 1-octen-3-ol, 5-methyl-2-furanmethanol, 6-methyl-5-hepten-2-ol, 1-nonen-4-ol, butanal, 2-methylbutanal, 3-methylbutanal, hexanal, furfurol, benzaldehyde, E-2-heptenal, E-2-octenal, n-nonanal, phenylacetaldehyde, pentanal, acetoin, 6-methyl-5-hepten-2-one, 1-octen-3-one, 2-pentanone, acetone, 3-octanone, 2-hexanone, 2-heptanone, methyl acetate, ethyl acetate, ethyl propanoate, propyl acetate, ethyl 2-hydroxypropanoate, ethyl 3-methylbutyrate, butyl acetate, isoamyl acetate, ethyl pentanoate, butyrolactone, ethyl hexanoate, methyl myristoleate, methyl hexanoate, ethyl benzoate, methyl benzoate, ethyl heptanoate, hexyl butanoate, gamma-terpinene, alpha-phellandrene, myrcene, limonene, 2-acetylfuran, 2-pentyl furan, o-cymene, and 2-cyclohexenone were purchased from MilliporeSigma (St. Louis, MO, USA).

Malic acid, citric acid, quinic acid, lactic acid, tartaric acid, glucose, fructose, maltose, sodium hydroxide, and potassium hydroxide were of HPLC grade (Sinopharm Chemical Reagent Co., Ltd., Beijing, China). Glutamic acid (GLU) and aspartic acid (ASP) were obtained from MilliporeSigma; serine (SER), threonine (THR), aspartic acid (ASP), glycine (GLY), alanine (ALA), citrulline (CIT), valine (VAL), methionine (MET), isoleucine (ILE), leucine (LEU), tyrosine (TYR), lysine (LYS), histidine (HIS), arginine (ARG), and proline (PRO) were purchased from Wako Pure Chemical Industries, Ltd. (Osaka, Japan).

### 2.3. Sample Preparation

The detailed procedure of sample preparation was referred to by Chen et al. (2018) [12]. Approximately 100 g of red jujubes were washed with running water and the moisture on the surface was wiped with filter papers. The kernel was removed, and the red jujubes were sliced and ground for 90 s with a juicer (JYL-CO20, Joyoung Co., Ltd., Jinan, China). A weight of 2.0 g red jujube pulp was placed into a 20 mL vial, which was sealed with a magnetic screw cap and septum before E-nose or GC-IMS testing.

### 2.4. E-nose Analysis

A commercial PEN 3.5 E-nose (Airsense Analytics, GmBH, Schwerin, Germany) containing ten metal oxide semiconductors (MOS) (Table S2) was used to distinguish among the overall aroma perception of the six cultivars of red jujubes. The samples were firstly equilibrated at room temperature (26 °C) for 30 min. The gaseous compounds in the headspace were pumped through a Teflon tube into the sensor arrays at 400 mL/min using reference air (filtered through charcoal). This clean air was also used to rinse the system at a flow rate of 600 mL/min. The cleaning time, zero adjustment time, and detection time were 180 s, 10 s, and 60 s, respectively. During the monitoring of the sample gas, the ratio of G (the conductance of a sensor exposed to sample gas) to G0 (the conductance of a sensor exposed to zero gas) for each sensor changed. WinMuster Software was used for data processing. Five repeats from the same 100 g sample were prepared.

### 2.5. HS-GC-IMS Analysis

A GC-IMS (GC from Agilent Technologies, Palo Alto, CA, USA; IMS from FlavourSpec^®^, Gesellschaft für Analytische Sensorsysteme mbH, Dortmund, Germany) system equipped with an autosampler unit (CTC Analytics AG, Zwingen, Switzerland) was used in this project. The vials prepared as in Section 2.3 were incubated at 50 °C for 20 min. Then a headspace volume of 500 μL was collected from the vials with a heated syringe (85 °C) and injected at 80 °C in splitless mode. GC conditions were as follows: the chromatographic column was a FS-SE-54-CB-1 (15 m × 0.53 mm ID × 1.0 µm d_f_) column (60 °C isothermal conditions). This is because the FS-SE-54-CB-1 column has also been used for the analysis of the volatile organic compounds in winter jujube and avocado based on the comprehensive separation effect (a better separation effect, a shorter separation time, and the boiling points, polarities, and species of VOCs) [15,16]. The program for carrier gas (nitrogen, 99.999%) was as follows: 2 mL/min for 2 min, ramped up to 10 mL/min for 8 min, then when 100 mL/min was reached for 10 min, finally altered to 150 mL/min for 5 min. All the standards that are presented in Section 2.1 were run under the same test procedure to support the GC-IMS Library Search for qualitative analysis. For the semi-quantitative analysis, 2-cyclohexenone (2 mg/L) was selected as the internal standard.

IMS conditions were as follows: ion source was a tritium source (5.68 keV), positive ion mode, drift tube length 9.8 cm, tube linear voltage 500 V/cm, and drift gas flow rate 150 mL/min (nitrogen, purity 99.999%). The temperature of the drift tube was 45 °C.

The spectrogram was elaborated using Laboratory Analytical Viewer (LAV). The Reporter Plug-In software was used to compare spectrogram differences between samples. Gallery Plot Plug-In was used to compare the differences in volatile fingerprints visually and quantitatively. Three technical repeats were conducted for each cultivar.

### 2.6. Sensory Analysis

The sensory analysis was conducted according to the method set by Galindo et al. (2015) [17]. The sensory analysis aimed to evaluate the following sensory attributes: sour, sweet, bitter, astringent, jujube ID, fruity, and floral [15]. The intensities of the various sensory attributes were evaluated using a numerical scale, where 0 represented none and 10 represented extremely strong, with 0.1 increments. A panel of twenty trained assessors (aged 21–30, ten females and ten males) was invited to evaluate the flavor of red jujubes. The assessors were recruited from fruit and vegetable processing teams and had extensive experience in fruit sensory evaluation. For the evaluation, 15 red jujubes for each cultivar per panelist were served in the testing room at 26 °C. There was a 10 min wait among the sensory evaluation for different cultivars.

### 2.7. Analysis of Fatty Acids, Amino Acids, Monosaccharides, and Organic Acids

The determination of fatty acids used the method reported by Song et al. (2019), using a GC–flame ionization detector (FID) (GC6890N, Agilent, Santa Clara, CA, USA). Undecanoic acid (5 mg/mL) was used as internal standard and the results were expressed in mg/g dry basis [5]. Analyses were carried out in triplicate.

The determination of free amino acids used the method reported by Song et al. (2019), using a Hitachi model L-8900 amino acid analyzer (Hitachi Co. Ltd., Tokyo, Japan) with a column packed with Hitachi custom ion-exchange resin 2622 (4.6 mm × 60 mm, particle size 5 μm).

Quantitative analyses of monosaccharides and organic acids in red jujubes were performed by high-performance anion-exchange chromatography equipped with pulsed amperometric detection (Dionex ICS-3000 system) according to Šimkovic et al. (2009) [14].

The standards of sugars and organic acids were tested under the same conditions for quantitative analysis using external calibrations.

### 2.8. Statistical Analysis

MetaboAnalyst (version 5.0, Edmonton, AB, Canada) (www.metaboanalyst.ca) was used for the calculation of VIP scores for VOCs. R (version 2.13.0) (Auckland, New Zealand) was used for the loading plot of principle component analysis (PCA) and correlation analysis between sensory attributes and chemical compositions. Each sample was analyzed in triplicate except for E-nose analysis, which had five replicates.

## 3. Results and Discussion

### 3.1. E-nose Analysis

**Figure 1:**
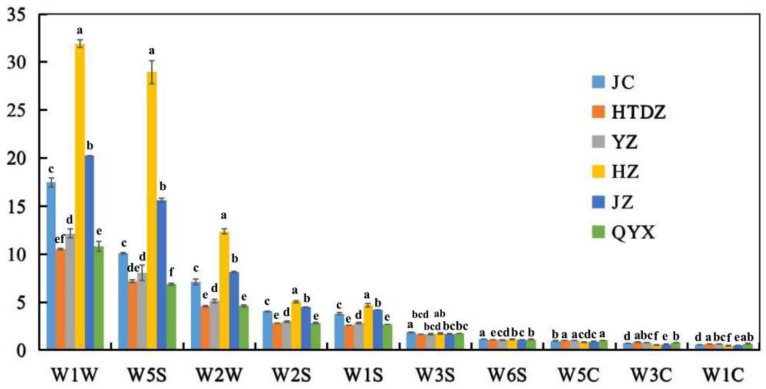
The response data of sensors in E-noses on volatiles of the six red jujube cultivars in Xinjiang Province, China (Jinchang—‘JC’, Junzao—‘JZ’, Huizao—‘HZ’,Qiyuexian—‘QYX’, Hetiandazao—‘HTDZ’, and Yuanzao—‘YZ’). Lower letters (a–f) correspond to significant differences at *p* < 0.05.

The averaged response values of different red jujube samples on ten MOS sensors are reported in Figure 1. The response values showed a relative standard deviation (RSD) lower than 5% (Table S2). The response signals for different red jujube samples increased at different rates. The most significant increase and the highest level in response signals were found in W1W sensitive to sulfides (minimum and maximum were 10.5~31.9), followed by W5S sensitive to nitroxide (6.0~29.0), W2W sensitive to aromatic components and organic sulfide (4.6~12.4), W2S (2.8~5.0) and W1S sensitive to methane (2.6~4.7). However, few responses were shown in the sensors, including W6S, W3C, W5C, W3S, and W1C (around 1.0), sensitive to hydrogen, ammonia and aromatic components, alkanes and aromatics, alkanes, and aromatics, respectively. The response values of W1W (31.9), W5S (29.0), and W2W (12.4) were notably higher in HZ, followed by JZ (W1W, 20.2; W5S, 15.6; W2W, 8.1), JC (W1W, 17.5; W5S, 10.1; W2W, 7.1) and YZ (W1W, 12.2; W5S, 8.1; W2W, 5.1). As shown in Figure 1, QYX and HTDZ showed the same response level in the sensors of W1W, W2W, W2S, W1S, W3S, and W5C, compared with other cultivars.

**Figure 2:**
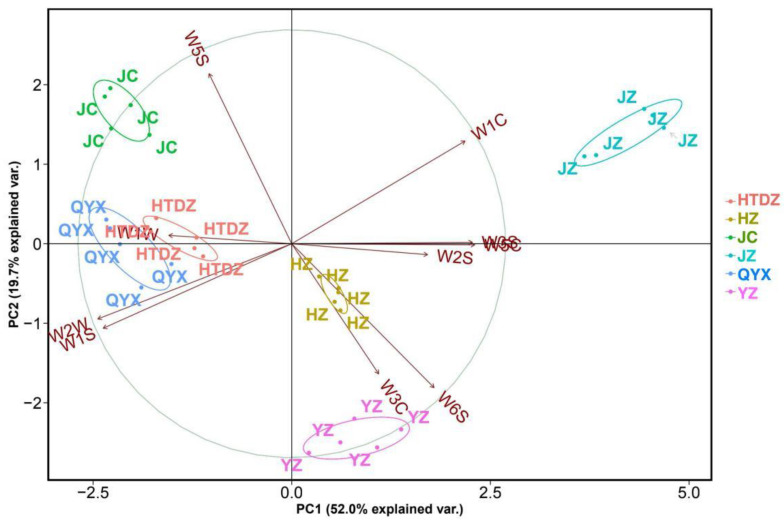
Principle component analysis (PCA) and loading plot of E- nose data for the six red jujube cultivars in Xinjiang Province, China (Jinchang—‘JC’, Junzao—‘JZ’, Huizao—‘HZ’,Qiyuexian—‘QYX’, Hetiandazao—‘HTDZ’, and Yuanzao—‘YZ’).

The data were processed using principal component analysis (PCA) to visualize whether the E-nose measurement was able to distinguish between the different red jujube cultivars. The sum of PC1 and PC2 accounts for 71.7% of the E-nose results (Figure 2). The technical repeats for the six cultivars were well clustered, except for a slight overlap of the HTDZ and QYX cultivars. The response value on W1W played an important role in the clustering of HTDZ and QYX (Figure 2). Meanwhile, the separation of JZ was mainly caused by the response value of W1C.

E-nose cannot provide qualitative information on the specific chemical compounds responsible for the observed discrimination, but when properly trained and validated with more information-rich techniques (e.g., GC-IMS), it could be used as a valid routine method to discriminate the different jujube varieties.

### 3.2. GC-IMS Analysis

The chromatograms of GC-IMS are shown in Figure S1. Sixty-four compounds were identified by GC-IMS (Table S3) in the six cultivars of red jujubes, including esters (17), acids (14), aldehydes (11), ketones (8), alcohols (6), terpenoids (6), and furans (2). Sixteen compounds, including hexanoic acid, isobutyric acid, 3-methylbutanoic acid, n-decanoic acid, heptanoic acid, 1-octen-3-ol, hexanal, benzaldehyde, E-2-heptenal, n-nonanal, 6-methyl-5-hepten-2-one, ethyl hexanoate, ethyl benzoate, methyl benzoate, 2-pentyl furan, and o-cymene, were reported in previous studies [18].

**Figure 3:**
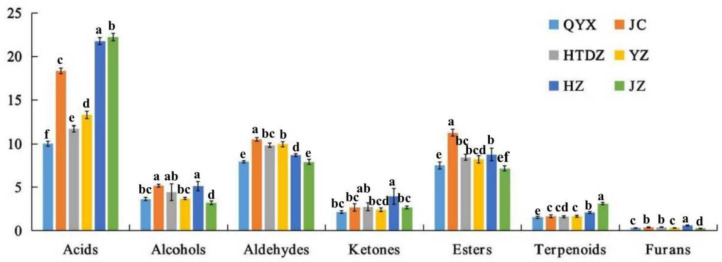
Semi-quantitative results of GC-IMS analysis of the volatiles in six red jujube cultivars taken from Xinjiang Province, China (Jinchang—‘JC’, Junzao—‘JZ’, Huizao—‘HZ’,Qiyuexian—‘QYX’, Hetiandazao—‘HTDZ’, and Yuanzao—‘YZ’). Lower letters (a–f) correspond to significant differences at *p* < 0.05.

**Figure 4:**
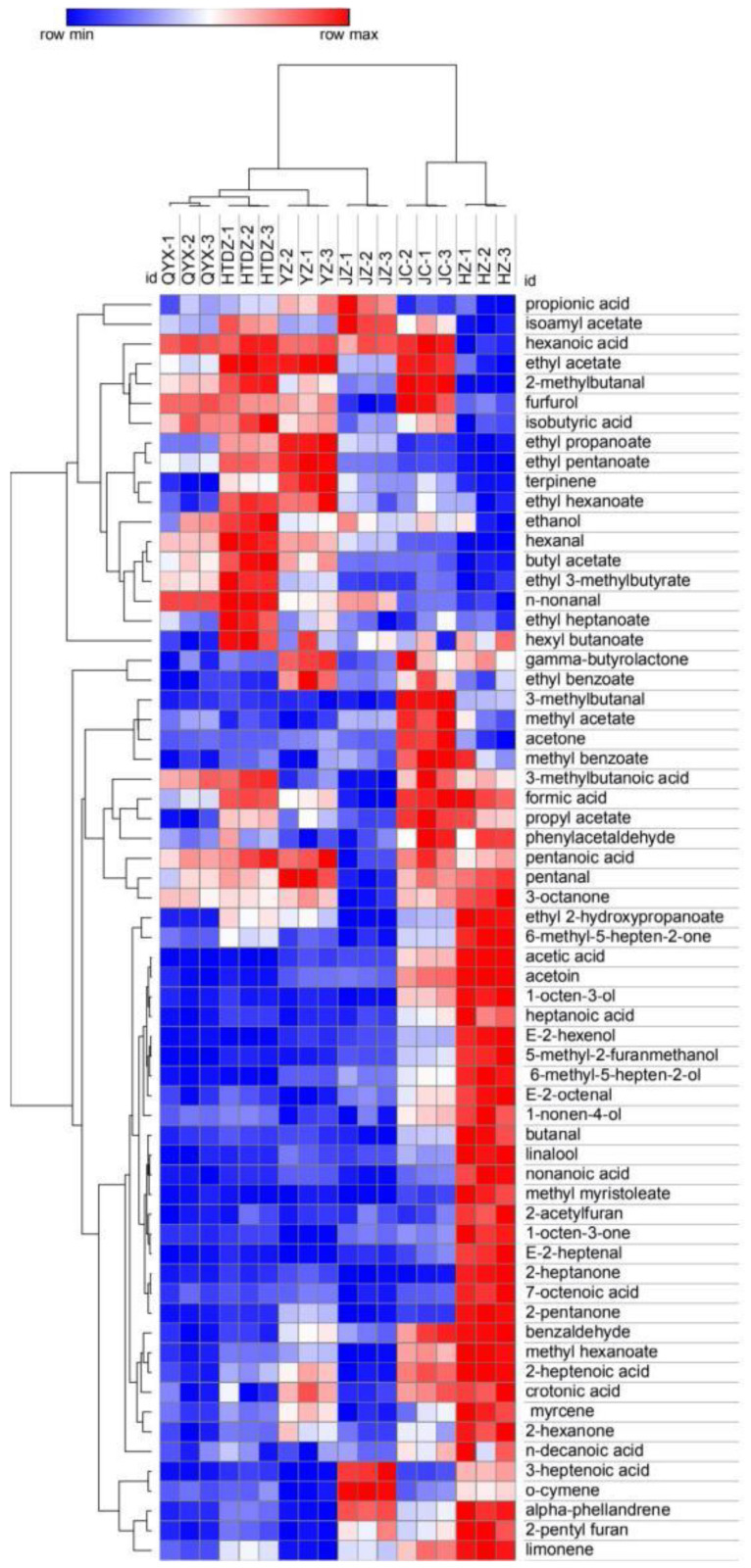
Heatmap analysis of volatiles taken from GC-IMS of the six red jujube cultivars in Xinjiang Province, China (Jinchang—‘JC’, Junzao—‘JZ’, Huizao—‘HZ’,Qiyuexian—‘QYX’, Hetiandazao—‘HTDZ’, and Yuanzao—‘YZ’).

The overall distribution of the different chemical classes according to the cultivar is reported in Figure 3. The 64 compounds identified were used to perform a hierarchical cluster analysis and the results were visualized using a heatmap (Figure 4). All the cultivars were well discriminated. The proximity of QYX and HTDZ was confirmed, although they were separated in the hierarchical cluster analysis. Overall, acids (formic acid, hexanoic acid, propionic acid, acetic acid, isobutyric acid, 3-methyl butanoic acid, pentanoic acid, 3-heptenoic acid, crotonic acid, nonanoic acid, 7-octenoic acid, n-decanoic acid, heptanoic acid, and 2-heptenoic acid), esters (isoamyl acetate, ethyl acetate, ethyl propanoate, ethyl pentanoate, ethyl hexanoate, butyl acetate, ethyl 3-methylbutyrate, ethyl heptanoate, hexyl butanoate, propyl acetate, and ethyl 2-hydroxypropanoate), and aldehydes (butanal, 2-methylbutanal, 3-methylbutanal, hexanal, furfurol, benzaldehyde, E-2-heptenal, E-2-octenal, n-nonanal, phenylacetaldehyde, and pentanal) were the more predominant VOCs of the six red jujube cultivars (Figure 3, Table S3).

HZ had the highest content of ketones (3.96 μg/g), including acetoin, 1-octen-3-one, 6-methyl-5-hepten-2-one, acetone, 2-hexanone, 2-pentanone, and 3-octanone. Acetoin acts as an aglycone and exists in a glycosidically bound form in some fruits and vegetables (banana, lychee, durian and kiwi fruit flowers, etc.), with a pleasant yogurt creamy odor [19]. Further, 1-octen-3-one was a result of the degradation of fatty acids, such as linoleic and linolenic acids, which also existed in red jujube [5,20]. It was noticed that 6-methyl-5-hepten-2-one, with a fruity and apple-like odor, also existed in the odor profile of tea [21]. Furthermore, the concentrations of linalool (0.16 μg/g), nonanoic acid (1.24 μg/g), methyl myristoleate (0.58 μg/g), 2-acetylfuran (0.54 μg/g), 1-octen-3-one (0.08 μg/g), (E)-2-heptenal (0.44 μg/g), 2-heptenone (1.00 μg/g), 7-octenoic acid (0.16 μg/g), and 2-pentanone (0.84 μg/g) were the highest in HZ. Linalool (3, 7-dimethyl-1, 6-octadien-3-ol) widely existed in plants, presenting a floral scent [22]. Furans were labeled by the caramel-like, sweet, fruity, and nutty odor descriptions [23]. In particular, 2-acetylfuran had a sweet, almond, and cream odor [23]. The (E)-2-alkenals, including (E)-2-heptenal, have been reported to contribute to the oxidative off flavor [24]. Further, 2-heptanone was described as one of the characteristic aroma compounds of ripe fruit, with a cinnamon and sweet odor [25]. JC was more abundant in the content of acetone (1.16 μg/g), methyl acetate (2.20 μg/g), 3-methyl butanal (0.77 μg/g), methyl benzoate (0.12 μg/g), and propyl acetate (2.42 μg/g). Acids were dominant in both JZ (22.22 μg/g) and YZ (13.27 μg/g).

QYX and HTDZ share a number of key features in the composition of VOCs. Acids (10.02 and 11.71 μg/g) accounted for the highest proportion in the aroma composition, followed by aldehydes (7.93 and 9.83 μg/g), esters (7.51 and 8.45 μg/g), alcohols (3.66 and 4.45 μg/g), ketones (2.14 and 2.75 μg/g), terpenoids (1.54 and 1.60 μg/g), and furans (0.31 and 0.40 μg/g) in QYX and HTDZ, respectively (Figure 3, Table S3). The concentrations of thirteen kinds of common VOCs in QYX and HTDZ were also comparable, including pentanoic acid (2.16 and 2.83 μg/g), ethanol (2.31 and 3.00 μg/g), isobutyric acid (0.58 and 0.55 μg/g), butyl acetate (0.54 and 0.61 μg/g), hexanoic acid (both 0.26 μg/g), 3-methyl butanoic acid (0.14 and 0.13 μg/g), hexanal (0.19 and 0.25 μg/g), ethyl 3-methylbutyrate (both 0.03 μg/g), n-nonanal (0.41 and 0.44 μg/g), furfurol (0.19 and 0.22 μg/g), 2-methyl butanal (0.53 and 0.61 μg/g), 3-octanone (both 0.03 μg/g), and pentanal (0.02 and 0.03 μg/g). Among these common VOCs, hexanoic acid and pentanoic acid were also found in grape and were perceived as having a sweat odor, n-nonanal as citrus with a green odor, and furfurol as a sweet odor [26]. Further, 3-methylbutanoic acid was identified as one of the most important odorants in chocolate, with fruity, sweaty, rancid, and cheesy aromas [27,28]. Isobutyric acid is always detected in milk products, with rancid, butter, cheese-like odors [29]. Ethanol was presented in food products with odor descriptions such as alcohol, floral, ripe apple, and sweet [30]. The presence of esters such as ethyl 3-methylbutyrate and butyl acetate always contributed to the fruity notes [31].

**Figure 5:**
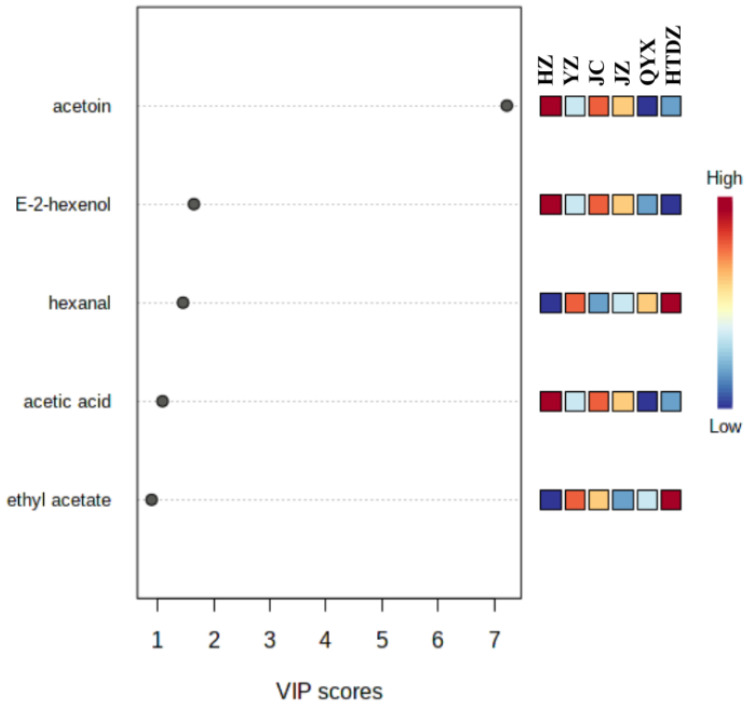
Volatiles with variable importance in projection (VIP) scores over 1 in the six red jujube cultivars from Xinjiang Province, China (Jinchang—‘JC’, Junzao—‘JZ’, Huizao—‘HZ’,Qiyuexian—‘QYX’, Hetiandazao—‘HTDZ’, and Yuanzao—‘YZ’).

The VIP scores were used to highlight the most discriminatory features that contribute to the clustering of the different cultivars. Acetoin, E-2-hexanol, hexanal, acetic acid, and ethyl acetate were the components with VIP score > 1, indicating their role in the discrimination of the different cultivars (Figure 5).

### 3.3. Fatty Acids, Amino Acids, Organic Acids, and Sugars

The composition in fatty acids, amino acids, organic acids, and sugars are reported in Supplementary Table S4. The content of organic acids, including malic acid, citric acid, quinic acid, lactic acid, and tartaric acid, in the six cultivars of red jujube was in the range of 22.87~159.01 mg/g. The amount of three kinds of sugars (glucose, fructose, and maltose) was 20.59~126.02 mg/g. The concentration of fatty acids and amino acids was in trace amounts (0~0.51 mg/g), except for proline (1.55~2.25 mg/g).

### 3.4. Correlation Analysis between Sensory Attributes and Chemical Composition

The sensory profiles of the six red jujube cultivars are displayed in Figure S2. All the six red jujube cultivars were characterized by higher values on jujube ID (a sweet and fruity flavor associated with jujube) (7.0~8.0), sweet (6.4~7.8), fruity attributes (5.8~6.4). The sour (1.9~3.2), floral (0.6~1.5), bitter (0.4~0.8), and astringent attributes (0.3~1.0) were relatively lower in the red jujubes, compared with the aforementioned three attributes. Additionally, HZ was more prominent in fruity (6.4) and floral (1.5) attributes. JZ had a higher value in sweet (7.8), sour (3.2), and jujube ID (8.0) attributes. HTDZ obtained higher scores in the bitter (0.8) and astringent (1.0) attributes. Furthermore, JC, QYX, and YZ showed higher values in jujube ID (8.0), sweet (7.2), and sour (3.0), respectively.

**Figure 6:**
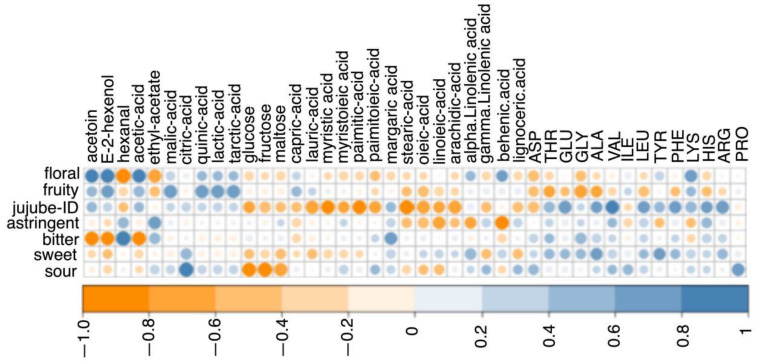
Correlation analysis among sensory attributes and fatty acids, amino acids, organic acids, sugars and VOCs (VIP score > 1) of the six red jujube cultivars from Xinjiang Province, China.

A correlation analysis between sensory characteristics and all VOCs, fatty acids, amino acids, organic acids, and sugars was also carried out. A redundancy analysis (RDA) was carried out to analyze the relationship between chemistry composition (factors) and sensory values. The correlation between the sensory profile and the chemical composition (limited to VOCs with VIP score > 1, as they played an important role in the differentiation of the cultivars) is reported in Figure 6, while the correlation with all the VOCs is reported in Supplementary Figure S3. The floral attribute was highly correlated with VOCs (acetoin, E-2-hexenol, and acetic acid). The fruity attribute was correlated with organic acids (malic acid, quinic acid, lactic acid, and tarctic acid). The jujube ID was mainly correlated with the amino acids (THR, GLU, GLY, ALA, VAL, LEU, TYR, PHE, LYS, HIS, and ARG). This finding could be correlated with the phenomenon that free amino acids may transaminate or dehydrogenate into aldehydes or acids [32]; for example, phenylacetaldehyde and isobutanoic acid could be issued from the oxidation of PHE and VAL, separately [33]. Thus, phenylacetaldehyde and isobutanoic acid may be contributors to the jujube ID. The concentration of MET and SER in fresh jujube (Junzao cultivar) at red maturity were 0.58 mg/100 g and 36.91 mg/100 g, respectively [5]. However, MET and SER were not detected in the six cultivars of red jujubes, suggesting that they might gradually decompose completely and form sulfur-containing substances, amine compounds, aldehydes, and ketones during the natural drying process (from fresh jujubes to red jujubes), through deamination and decarboxylation reactions. The astringency attribute was only correlated with hexanal and ethyl acetate. Astringency was perceived as a comprehensive feeling of roughing, drying, shrinking or drawing, which could increase and last for a longer time after swallowing [34]. Phenolic components, multivalent salts, such as alum, organic acids, and charged polysaccharides, such as chitosan, can stimulate astringency [35,36]. The astringent attribute is, thus, a complex physicochemical reaction among volatile chemicals, non-volatile chemicals, and chewing, thus explaining the low correlation with the compounds examined here. The bitter attribute was correlated with hexanal and margaric acid. The sweet attribute covaried with amino acids (THR, GLU, GLY, ALA, VAL, LEU, TYR, PHE, LYS, HIS, and ARG). Amino acids were perceived as one or more tastes according to their structures. In the cases of L-/D-ARG and L-/D-HIS, the main taste was evaluated as sweet and/or bitter. The cases of GLY, THR, and ALA were expected to be sweet in red jujubes. VAL, LEU, and PHE were evaluated as sweet and bitter tastes in D-form [37]. The sour attribute had a close relationship with organic acids (malic acid, citric acid, quinic acid, lactic acid, and tartaric acid), amino acids (ILE and PRO), and some fatty acids. Among the five correlated organic acids, citric acid could contribute to a sour gustatory flavor quality, as in lemon [38]. However, L-ILE and D-PRO were evaluated as a bitter taste [37]. As a result, the taste was a complex effect of the structural properties of amino acids, which could be explained by the fact that different structures of amino acids were perceived by multiple taste receptors [39,40].

## 4. Conclusions

Volatile profiles of six red jujube cultivars, which originated from Xinjiang Province, China, were analyzed by E-nose and GC-IMS. The chemical compositions of the non-volatile metabolites, i.e., fatty acids, amino acids, organic acids, and sugars, were assessed, and a sensory evaluation was also performed. Descriptive sensory analysis and multivariate data analysis were carried out to correlate the sensory perception with the chemical fingerprinting. A total of sixty-four kinds of VOCs have been identified in the six red jujube cultivars (HZ, JZ, YZ, JC, HTDZ, and QYX). Esters and acids were the more predominant VOCs. Acetoin, E-2-hexanol, hexanal, acetic acid, and ethyl acetate played an important role for the discrimination of the six red jujube cultivars. The differences in sensory attributes of the red jujubes could be explained by the composition of VOCs, amino acids, fatty acids, and organic acids, to some extent.

## Data Availability

Data is available upon request.

## Supplementary Materials

The following supporting information can be downloaded at: https://www.mdpi.com/article/10.3390/foods11030421/s1: Figure S1: the chromatograms of the six red jujube cultivars in Xinjiang Province, China (Jinchang—‘JC’, Junzao—‘JZ’, Huizao—‘HZ’,Qiyuexian—‘QYX’, Hetiandazao—‘HTDZ’, and Yuanzao—‘YZ’); Figure S2: sensory profiles of the six red jujube cultivars in Xinjiang Province, China (Jinchang—‘JC’, Junzao—‘JZ’, Huizao—‘HZ’,Qiyuexian—‘QYX’, Hetiandazao—‘HTDZ’, and Yuanzao—‘YZ’); Figure S3: correlation analysis among sensory attributes and VOCs of the six red jujube cultivars from Xinjiang Province, China (Jinchang—‘JC’, Junzao—‘JZ’, Huizao—‘HZ’,Qiyuexian—‘QYX’, Hetiandazao—‘HTDZ’, and Yuanzao—‘YZ’); Table S1: information about six cultivars of red jujube in Xinjiang Province, China; Table S2: response characteristics and repeatability of response values of ten sensors in E-nose; Table S3: composition identification of volatiles in six red jujube cultivars from Xinjiang Province, China, via GC-IMS; Table S4: the composition of fatty acids, amino acids, organic acids, and sugars of six red jujube cultivars from Xinjiang Province, China.
