# Supplementary material for: Investigation of the Volatile Profile of Red Jujube by Using GC-IMS, Multivariate Data Analysis, and Descriptive Sensory Analysis
Source: Foods. 2022 Jan 31;11(3):421. doi: 10.3390/foods11030421 (PMC8834224; doi:10.3390/foods11030421)
Supplement: Supplementary file 1 [file foods-11-00421-s001.zip › foods-1542302-supplementary.pdf]

**Supplementary material**

**Table S1** Information about six cultivars of red jujube in Xinjiang Province, China

| Code | Cultivar | Origin | Traits | | |
|---|---|---|---|---|---|
| QYX | Qiyuexian | Xinjiang, Akesu | 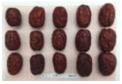 | 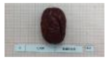 | 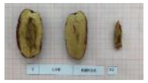 |
| JC | Jinchang | Xinjiang, Akesu | 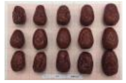 | 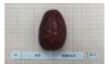 | 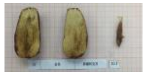 |
| HTDZ | Hetiandazao | Xinjiang, Khotan | 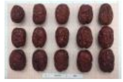 | 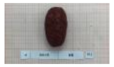 | 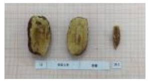 |
| YZ | Yuanzao | Xinjiang, Hami | 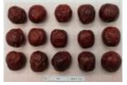 | 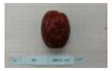 | 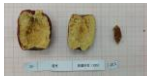 |
| HZ | Huizao | Xinjiang, Charkhlik | 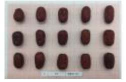 | 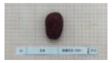 | 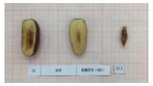 |
| JZ | Junzao | Xinjiang, Khotan | 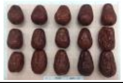 | 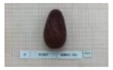 | 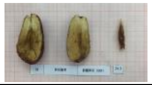 |

Note: Jinchang—'JC', Junzao—'JZ', Huizao—'HZ',Qiyuexian—'QYX', Hetiandazao—'HTDZ', and Yuanzao—'YZ'.

**Table S2** Response characteristics and repeatability of response values of ten sensors in E-nose

| Number | Sensors | Response characteristics | RSD% |
|---|---|---|---|
| 1 | W1C | aromatic compounds | 0.18 |
| 2 | W5S | nitroxide | 4.54 |
| 3 | W3C | ammonia and aromatic components | 0.28 |
| 4 | W6S | hydrogen selective | 0.85 |
| 5 | W5C | alkanes and aromatic components | 1.65 |
| 6 | W1S | methane | 0.81 |
| 7 | W1W | sulfides | 0.65 |
| 8 | W2S | ethyl alcohol | 0.74 |
| 9 | W2W | aromatic components and organic sulfide | 0.59 |
| 10 | W3S | alkanes | 0.56 |

RSD: relative standard deviation

**Table S3** The GC-IMS results of the volatile composition of the six red jujube cultivars.

| | Compound | RI | Rt [sec] | Dt [a.u.] | Concentration(μg/g) | | | | | |
|---|---|---|---|---|---|---|---|---|---|---|
| | | | | | Qiyuexian (QYX) | Jinchang (JC) | Hetianda zao (HTDZ) | Yuanzao (YZ) | Huizao (HZ) | Junzao (JZ) |
| Acids（14） | formic acid | 568.30 | 130.64 | 1.16 | 0.30±0.08c | 0.27±0.07a | 0.32±0.03a | 0.37±0.07b | 0.26±0.14a | 0.40±0.05d |
| | hexanoic acid | 576.70 | 134.37 | 1.18 | 0.26±0.02ab | 1.01±0.04a | 0.26±0.08ab | 0.40±0.04b | 1.47±0.05c | 0.42±0.11b |
| | propionic acid | 659.50 | 170.83 | 1.44 | 0.05±0.02bc | 0.08±0.01cd | 0.06±0.01b | 0.07±0.02a | 0.18±0.02d | 0.05±0.03a |
| | acetic acid | 659.90 | 170.99 | 1.48 | 0.04±0.01d | 0.06±0.04b | 0.04±0.01d | 0.06±0.04c | 0.07±0.01a | 0.04±0.01c |
| | isobutyric acid | 749.20 | 234.36 | 1.56 | 0.58±0.02ab | 1.26±0.02b | 0.55±0.02a | 0.73±0.01b | 2.52±0.02c | 0.83±0.01c |
| | 3-methyl butanoic acid | 749.20 | 234.36 | 1.45 | 0.14±0.02ab | 0.37±0.05a | 0.13±0.02a | 0.22±0.04c | 0.62±0.02b | 0.27±0.01c |
| | pentanoic acid | 844.30 | 333.33 | 1.61 | 2.16±0.03bc | 1.30±0.03ab | 2.83±0.05a | 2.26±0.05a | 0.96±0.03c | 1.73±0.04d |
| | 3-heptenoic acid | 1055.60 | 692.06 | 1.53 | 3.22±0.02d | 9.07±0.01c | 3.26±0.01c | 4.62±0.02d | 10.95±0.02b | 4.68±0.04a |
| | crotonic acid | 807.30 | 290.37 | 1.41 | 1.91±0.01c | 2.82±0.01b | 1.90±0.02c | 2.03±0.02b | 1.84±0.01a | 1.92±0.04c |
| | nonanoic acid | 1024.20 | 632.73 | 1.39 | 0.21±0.01b | 0.54±0.01a | 0.74±0.01b | 0.65±0.03a | 1.24±0.02a | 0.15±0.02b |
| | 7-octenoic acid | 905.90 | 414.10 | 1.23 | 0.04±0.01c | 0.08±0.00bc | 0.05±0.01bc | 0.09±0.01b | 0.16±0.01a | 0.06±0.01d |
| | n-decanoic acid | 887.30 | 383.31 | 1.56 | 0.05±0.01b | 0.06±0.01a | 0.08±0.01b | 0.07±0.02b | 0.07±0.01a | 0.06±0.03b |
| | heptanoic acid | 1219.70 | 1002.43 | 1.54 | 0.96±0.02b | 1.19±0.01a | 1.32±0.04b | 1.62±0.05b | 0.97±0.02a | 1.20±0.01b |
| | 2-heptenoic acid | 1012.90 | 611.23 | 2.11 | 0.10±0.01e | 0.25±0.01b | 0.17±0.01d | 0.08±0.02c | 0.46±0.04a | 0.41±0.01e |
| | Total | | | | 10.02±0.29f | 18.36±0.32c | 11.71±0.33e | 13.27±0.44d | 21.77±0.42b | 22.22±0.42a |
| Alcohols (6) | ethanol | 532.50 | 114.88 | 1.16 | 2.31±0.14b | 3.15±0.04ab | 3.00±0.03a | 2.53±0.02ab | 3.06±0.17a | 1.71±0.09b |
| | E-2-hexenol | 730.10 | 218.38 | 1.47 | 0.14±0.01e | 0.44±0.02b | 0.18±0.01e | 0.17±0.04d | 0.83±0.07a | 0.25±0.03c |
| | 1-octen-3-ol | 771.00 | 252.57 | 1.26 | 0.09±0.02c | 0.18±0.04b | 0.11±0.03c | 0.08±0.02c | 0.26±0.02a | 0.12±0.01c |
| | 5-methyl-2-furanmethanol | 1041.70 | 665.70 | 1.14 | 0.77±0.01e | 1.14±0.02b | 0.66±0.01d | 0.63±0.01de | 0.78±0.04a | 0.80±0.01c |

| | | | | | | | | | | |
|---|---|---|---|---|---|---|---|---|---|---|
| | 6-methyl-5-hepten-2-ol | 650.60 | 166.89 | 1.28 | 0.19±0.00e | 0.1±0.01b | 0.28±0.00e | 0.15±0.01d | 0.07±0.02a | 0.09±0.03c |
| | 1-nonen-4-ol | 812.20 | 296.10 | 1.79 | 0.16±0.00cd | 0.19±0.03b | 0.22±0.02c | 0.15±0.02d | 0.11±0.00a | 0.24±0.05d |
| | Total | | | | 3.66±0.18bc | 5.20±0.16a | 4.45±0.97ab | 3.71±0.12bc | 5.11±0.54a | 3.21±0.22d |
| Aldehydes(11) | butanal | 563.50 | 128.57 | 1.22 | 0.54±0.01cd | 0.52±0.01b | 0.57±0.02c | 0.52±0.02c | 0.42±0.05a | 0.45±0.02d |
| | 2-methylbutanal | 642.20 | 163.20 | 1.20 | 0.53±0.00b | 0.59±0.00a | 0.61±0.01a | 0.62±0.02b | 0.51±0.03d | 0.30±0.01c |
| | 3-methylbutanal | 652.10 | 167.55 | 1.35 | 0.10±0.02cd | 0.77±0.01a | 0.15±0.04c | 0.09±0.06d | 0.48±0.02b | 0.65±0.04d |
| | hexanal | 742.10 | 228.36 | 1.26 | 0.19±0.01c | 0.37±0.01e | 0.25±0.07a | 0.32±0.07b | 0.42±0.01f | 0.17±0.07d |
| | furfurol | 735.80 | 223.09 | 1.09 | 0.19±0.00b | 0.38±0.02a | 0.22±0.01bc | 0.21±0.01c | 0.72±0.01d | 0.15±0.01e |
| | benzaldehyde | 815.40 | 299.81 | 0.95 | 0.03±0.03e | 0.06±0.08b | 0.04±0.03e | 0.03±0.04c | 0.17±0.01a | 0.04±0.05d |
| | E-2-heptenal | 973.50 | 538.12 | 1.11 | 0.08±0.01c | 0.07±0.01b | 0.10±0.03c | 0.12±0.01c | 0.44±0.01a | 0.06±0.01c |
| | E-2-octenal | 645.60 | 164.68 | 1.31 | 5.38±0.01de | 6.60±0.01b | 6.71±0.01cd | 6.70±0.01e | 4.46±0.01a | 5.12±0.01c |
| | n-nonanal | 890.40 | 386.83 | 1.11 | 0.41±0.02b | 0.58±0.03e | 0.44±0.01a | 0.62±0.02d | 0.55±0.01f | 0.43±0.01c |
| | phenylacetaldehyde | 729.40 | 217.77 | 1.44 | 0.46±0.01c | 0.54±0.01a | 0.71±0.01bc | 0.70±0.02c | 0.46±0.01ab | 0.52±0.03c |
| | pentanal | 1181.60 | 930.25 | 1.82 | 0.02±0.02d | 0.03±0.01bc | 0.03±0.01cd | 0.03±0.02a | 0.07±0.01ab | 0.03±0.01e |
| | Total | | | | 7.93±0.14e | 10.51±0.20a | 9.83±0.25bc | 9.96±0.30b | 8.70±0.18d | 7.92±0.27e |
| Ketones (8) | acetoin | 705.00 | 197.35 | 1.23 | 0.08±0.02d | 0.09±0.01b | 0.08±0.01d | 0.10±0.04c | 0.24±0.03a | 0.06±0.01c |
| | 6-methyl-5-hepten-2-one | 772.50 | 253.78 | 1.34 | 0.29±0.01c | 0.18±0.02b | 0.31±0.01b | 0.23±0.02c | 0.15±0.01a | 0.26±0.02d |
| | 1-octen-3-one | 1059.50 | 699.44 | 1.22 | 0.03±0.03d | 0.05±0.02b | 0.04±0.01d | 0.06±0.02e | 0.08±0.01a | 0.04±0.01c |
| | 2-pentanone | 1022.70 | 629.87 | 1.23 | 0.29±0.01d | 0.66±0.01c | 0.39±0.01c | 0.46±0.01b | 0.84±0.01a | 0.25±0.01d |
| | acetone | 1016.50 | 618.22 | 1.90 | 0.76±0.02bc | 1.16±0.07a | 0.97±0.00bc | 0.65±0.06b | 1.04±0.20c | 0.94±0.02bc |
| | 3-octanone | 1017.80 | 620.55 | 1.20 | 0.03±0.03b | 0.05±0.04b | 0.03±0.02b | 0.02±0.04b | 0.08±0.05a | 0.06±0.03c |
| | 2-hexanone | 921.30 | 442.41 | 1.50 | 0.26±0.01d | 0.31±0.02b | 0.29±0.03c | 0.15±0.01b | 0.53±0.03a | 0.82±0.02cd |
| | 2-heptanone | 1015.20 | 615.62 | 1.68 | 0.40±0.01d | 0.18±0.03de | 0.64±0.01c | 0.76±0.01b | 1.00±0.01a | 0.24±0.04e |
| | Total | | | | 2.14±0.14bc | 2.68±0.42abc | 2.75±0.46ab | 2.43±0.21bcd | 3.96±0.89a | 2.67±0.16bc |

| | | | | | | | | | | |
|---|---|---|---|---|---|---|---|---|---|---|
| Esters (17) | methyl acetate | 545.70 | 120.69 | 1.19 | 2.10±0.03bc | 2.20±0.04ab | 2.15±0.03cd | 2.07±0.04d | 1.33±0.12b | 2.04±0.01b |
| | ethyl acetate | 583.80 | 137.48 | 1.24 | 0.58±0.09b | 0.60±0.06a | 0.63±0.06a | 0.39±0.06a | 0.53±0.30c | 0.33±0.03c |
| | ethyl propanoate | 733.10 | 220.87 | 1.39 | 0.04±0.01d | 0.11±0.01e | 0.06±0.02b | 0.05±0.02a | 0.16±0.01f | 0.05±0.02c |
| | propyl acetate | 767.10 | 249.34 | 1.23 | 1.46±0.02d | 2.42±0.06a | 1.67±0.01b | 1.42±0.01c | 1.26±0.01b | 1.46±0.05d |
| | ethyl 2-hydroxypropanoate | 767.70 | 249.84 | 1.54 | 0.35±0.03e | 0.89±0.02d | 0.37±0.05b | 0.36±0.07c | 1.20±0.01a | 0.32±0.01e |
| | ethyl 3-methylbutyrate | 746.10 | 231.70 | 1.48 | 0.03±0.01b | 0.04±0.01d | 0.03±0.01a | 0.03±0.01c | 0.05±0.01e | 0.03±0.00de |
| | butyl acetate | 759.40 | 242.88 | 1.12 | 0.54±0.03b | 0.63±0.03c | 0.61±0.01a | 0.52±0.01b | 0.40±0.02d | 0.46±0.03c |
| | isoamyl acetate | 779.20 | 259.43 | 1.08 | 0.85±0.05d | 2.00±0.01c | 0.89±0.01b | 0.82±0.04d | 1.22±0.03e | 0.81±0.01a |
| | ethyl pentanoate | 811.90 | 295.77 | 1.46 | 0.28±0.02c | 0.30±0.00e | 0.27±0.02b | 0.25±0.02a | 0.16±0.01f | 0.12±0.01d |
| | butyrolactone | 815.40 | 299.81 | 1.37 | 0.58±0.04b | 1.14±0.06a | 0.62±0.01b | 0.90±0.01a | 1.24±0.03a | 0.72±0.02b |
| | ethyl hexanoate | 899.10 | 401.57 | 1.34 | 0.02±0.02c | 0.04±0.03b | 0.03±0.02a | 0.02±0.04a | 0.03±0.06c | 0.02±0.05bc |
| | methyl myristoleate | 881.20 | 376.19 | 1.41 | 0.10±0.01c | 0.14±0.01b | 0.13±0.01c | 0.27±0.02c | 0.58±0.01a | 0.09±0.00c |
| | methyl hexanoate | 826.10 | 312.30 | 1.26 | 0.29±0.01e | 0.20±0.02b | 0.60±0.01d | 0.79±0.03c | 0.14±0.01a | 0.40±0.01f |
| | ethyl benzoate | 880.50 | 375.38 | 1.33 | 0.05±0.01b | 0.11±0.01a | 0.09±0.01b | 0.07±0.01a | 0.09±0.01b | 0.06±0.01b |
| | methyl benzoate | 759.30 | 242.76 | 1.38 | 0.01±0.00c | 0.12±0.01a | 0.01±0.00c | 0.01±0.02c | 0.10±0.04b | 0.01±0.01bc |
| | ethyl heptanoate | 766.60 | 248.85 | 1.43 | 0.15±0.02b | 0.25±0.01b | 0.17±0.01a | 0.15±0.01b | 0.21±0.01b | 0.17±0.01b |
| | hexyl butanoate | 765.20 | 247.72 | 1.47 | 0.08±0.01c | 0.08±0.01bc | 0.12±0.06a | 0.09±0.01b | 0.07±0.03ab | 0.07±0.01bc |
| | Total | | | | 7.51±0.41e | 11.27±0.40a | 8.45±0.34bc | 8.21±0.43bcd | 8.77±0.72b | 7.16±0.29ef |
| Terpenoids (6) | gamma-terpinene | 693.40 | 187.66 | 1.29 | 0.04±0.03d | 0.05±0.05c | 0.05±0.03b | 0.04±0.05a | 0.06±0.02d | 0.05±0.07c |
| | alpha-phellandrene | 1055.90 | 692.69 | 1.19 | 0.18±0.02e | 0.22±0.01c | 0.20±0.01d | 0.26±0.01f | 0.24±0.02a | 0.09±0.01b |
| | myrcene | 909.60 | 420.86 | 1.37 | 0.06±0.02de | 0.10±0.01c | 0.06±0.01cd | 0.04±0.00b | 0.25±0.03a | 0.09±0.01f |
| | limonene | 810.50 | 294.08 | 1.69 | 0.11±0.02d | 0.08±0.06b | 0.15±0.02c | 0.12±0.01e | 0.06±0.02a | 0.08±0.02c |
| | linalool | 766.50 | 248.84 | 1.56 | 0.04±0.01d | 0.08±0.01b | 0.05±0.01d | 0.06±0.01c | 0.16±0.01a | 0.05±0.01c |
| | o-cymene | 783.00 | 262.64 | 1.41 | 1.11±0.02c | 1.15±0.03c | 1.09±0.04c | 1.15±0.02d | 1.33±0.02b | 2.76±0.02a |

| | | | | | | | | | | |
|---|---|---|---|---|---|---|---|---|---|---|
| | Total | | | | 1.54±0.12de | 1.68±0.17c | 1.60±0.12cd | 1.67±0.10c | 2.10±0.12b | 3.12±0.14a |
| | 2-acetylfuran | 970.60 | 532.87 | 1.22 | 0.29±0.01b | 0.36±0.02b | 0.37±0.01b | 0.28±0.02b | 0.54±0.02a | 0.24±0.01b |
| Furans (2) | 2-pentyl furan | 1013.50 | 612.40 | 2.02 | 0.02±0.01d | 0.03±0.02b | 0.03±0.01c | 0.04±0.01d | 0.06±0.01a | 0.02±0.01b |
| | Total | | | | 0.31±0.02c | 0.39±0.04b | 0.40±0.02b | 0.32±0.03c | 0.60±0.03a | 0.26±0.02d |

Mean values with different letters in the same row correspond to significant differences at $p < 0.05$. Data are represented as the mean ± SD.

GC-IMS: gas chromatography ion mobility spectrometry.

‘The six red jujube cultivars’ includes Jinchang-‘JC’, Junzao-‘JZ’, Huizao-‘HZ’,Qiyuexian-‘QYX’, Hetiandazao-‘HTDZ’, and Yuanzao-‘YZ’.

‘RI’ means ‘retention index’, ‘Rt’ means ‘retention time’, ‘Dt’ means ‘drift time’.

**Table S4** The composition of fatty acids, amino acids, organic acids, and sugars of six red jujube cultivars from Xinjiang Province, China

| **Composition** | **Concentration(mg/g)** | | | | | |
|---|---|---|---|---|---|---|
| | Qiyuexian (QYX) | Jinchang (JC) | Hetiandazao (HTDZ) | Yuanzao (YZ) | Huizao (HZ) | Junzao (JZ) |
| malic acid | 105.38±22.54a | 106.01±18.34a | 131.57±33.67a | 145.47±43.23a | 154.50±21.34a | 159.01±54.42a |
| citric acid | 22.87±3.46c | 31.29±4.56bc | 25.75±6.76bc | 34.67±4.32b | 23.96±3.50c | 44.97±7.67a |
| quinic acid | 91.37±10.22a | 92.26±10.23a | 100.67±12.89a | 106.79±20.98a | 111.19±23.56a | 112.84±18.76a |
| lactic acid | 56.38±6.44c | 61.68±6.46c | 85.59±7.74b | 101.80±9.09ab | 116.45±89.76a | 121.84±20.03a |
| tartaric acid | 55.49±9.87d | 59.77±5.78d | 86.97±6.43c | 105.54±8.90b | 118.82±9.76ab | 123.57±12.65a |
| glucose | 126.02±13.45a | 84.70±7.65c | 108.37±9.80b | 87.22±6.73c | 96.93±7.83bc | 55.25±4.56d |
| fructose | 150.67±1.24a | 104.24±1.43d | 121.68±1.23b | 98.87±1.20e | 118.61±41.10c | 70.99±1.20f |
| maltose | 28.64±3.00a | 26.07±8.00a | 27.71±5.00a | 25.04±2.13a | 26.15±2.23a | 20.59±1.80a |
| capric acid | 0.22±0.01c | nd | nd | 0.35±0.01a | 0.26±0.01b | 0.26±0.01b |
| lauric acid | 1.98±0.01b | 0.81±0.01e | 0.77±0.01f | 2.91±0.01a | 1.68±0.01c | 1.21±0.01d |
| myristic acid | 2.16±0.01b | 0.74±0.01e | 0.68±0.01f | 3.44±0.02a | 1.27±0.01c | 1.16±0.01d |
| myristoleic acid | 1.45±0.01b | 0.54±0.01e | 0.57±0.03d | 5.08±0.01a | 0.40±0.01f | 1.02±0.01c |
| palmitic acid | 12.30±0.01b | 4.44±0.01f | 4.67±0.01e | 1.74±0.01a | 5.47±0.01d | 7.13±0.01c |
| palmitoleic acid | 4.28±0.02b | 2.22±0.02e | 2.39±0.02d | 6.35±0.02a | 1.35±0.02f | 3.91±0.02c |
| margaric acid | nd | nd | 0.02±1.00E-4a | nd | nd | 0.18±0.03b |
| stearic acid | 2.32±0.03a | 0.84±0.03d | 0.73±0.03e | 0.14±0.03b | 0.76±0.03f | 0.94±0.03c |

| | | | | | | |
|---|---|---|---|---|---|---|
| oleic acid | 5.89±0.02a | 0.84±0.002e | 0.79±0.02e | 1.43±0.02d | 0.37±0.02c | 1.37±0.02b |
| linoleic acid | 3.46±0.01a | 0.71±0.01d | 0.69±0.01d | 0.77±0.01b | 1.04±0.01d | 1.52±0.01c |
| arachidic acid | 0.34±0.01b | nd | nd | 0.28±0.02c | nd | 0.21±0.01a |
| alpha-linolenic acid | 0.28±0.01b | 0.17±0.01d | 0.19±0.01d | 0.20±0.01d | 0.28±0.01c | 0.47±0.01a |
| gamma-linolenic acid | nd | nd | nd | 0.18±0.01a | nd | nd |
| behenic acid | 0.27±0.01d | nd | nd | nd | 0.26±0.01e | 0.31±0.01c |
| lignoceric acid | nd | nd | nd | 0.24±0.01a | nd | nd |
| ASP | 4.40E-2±2.00E-4c | 3.60E-2±2.00E-4b | 3.30E-2±2.00E-4a | 5.30E-2±2.00E-4e | 2.10E-2±2.00E-4f | 4.20E-2±2.00E-4d |
| THR | 0.28±1.00E-3d | 0.51±1.00E-3a | 0.48±1.00E-3b | 0.17±1.00E-3f | 0.19±1.00E-3e | 0.34±1.00E-3c |
| SER | nd | nd | nd | nd | nd | nd |
| GLU | 1.10E-2±3.00E-4c | 3.20E-2±3.00E-4e | 2.60E-2±3.00E-4e | 3.50E-3±3.00E-4b | 1.30E-2±3.00E-4d | 0.02±3.00E-4a |
| GLY | 4.90E-3±1.00E-4d | 5.50E-3±1.00E-4a | 5.90E-3±2.00E-4b | 3.10E-3±2.00E-4f | 3.00E-3±1.00E-4e | 0.0043±1.00E-4c |
| ALA | 1.50E-2±1.00E-4c | 2.80E-2±1.00E-4d | 2.60E-2±1.00E-4b | 5.10E-3±1.00E-4e | 8.00E-3±1.00E-4b | 2.30E-2±1.00E-4a |
| VAL | 5.30E-3±4.00E-4d | 4.20E-2±3.00E-4a | 3.70E-2±2.00E-4b | 0.01±2.00E-4e | 1.40E-2±4.00E-4d | 2.80E-2±1.00E-4c |
| MET | nd | nd | nd | nd | nd | nd |
| ILE | 0.03±1.00E-4d | 1.50E-2±1.00E-4b | 1.50E-2±1.00E-4a | 4.70E-2±1.00E-4e | 2.90E-2±1.00E-4c | 5.10E-2±1.00E-4b |
| LEU | 2.40E-3±1.00E-4c | 1.90E-2±1.00E-4e | 1.50E-2±1.00E-4e | 1.60E-3±1.00E-4b | 2.00E-3±1.00E-4d | 8.70E-3±1.00E-4a |
| TYR | 1.80E-2±2.00E-4d | 1.30E-2±2.00E-4a | 2.10E-2±2.00E-4b | 1.00E-3±2.00E-4f | 0.021±2.00E-4e | 0.04±2.00E-4c |
| PHE | 9.30E-3±1.00E-4c | 2.20E-2±1.00E-4c | 2.10E-2±2.00E-4b | 1.00E-3±1.00E-4c | 9.20E-3±2.00E-4b | 1.40E-3±1.00E-4a |
| LYS | 7.00E-4±1.00E-4d | 4.30E-3±3.00E-4a | 3.90E-3±2.00E-4b | nd | 2.50E-3±1.00E-4d | 3.90E-3±1.00E-4c |
| HIS | nd | 0.01±1.00E-4c | 9.30E-3±1.00E-4d | nd | nd | 3.10E-3±1.00E-4a |
| ARG | 2.30E-2±1.00E-4d | 4.70E-2±1.00E-4b | 5.80E-2±1.00E-4a | nd | 2.80E-2±1.00E-4c | 4.70E-2±1.00E-4b |

| | | | | | | |
|---|---|---|---|---|---|---|
| PRO | 1.70±0.12b | 1.64±0.12b | 1.55±0.12b | 2.25±0.12a | 1.66±0.12b | 2.17±0.12a |

nd: not detected. Mean values with different letters in the same row correspond to significant differences at $p < 0.05$. Data are represented as the mean ± SD.

'E' represents the corresponding power of '10' in the scientific notation of the table.

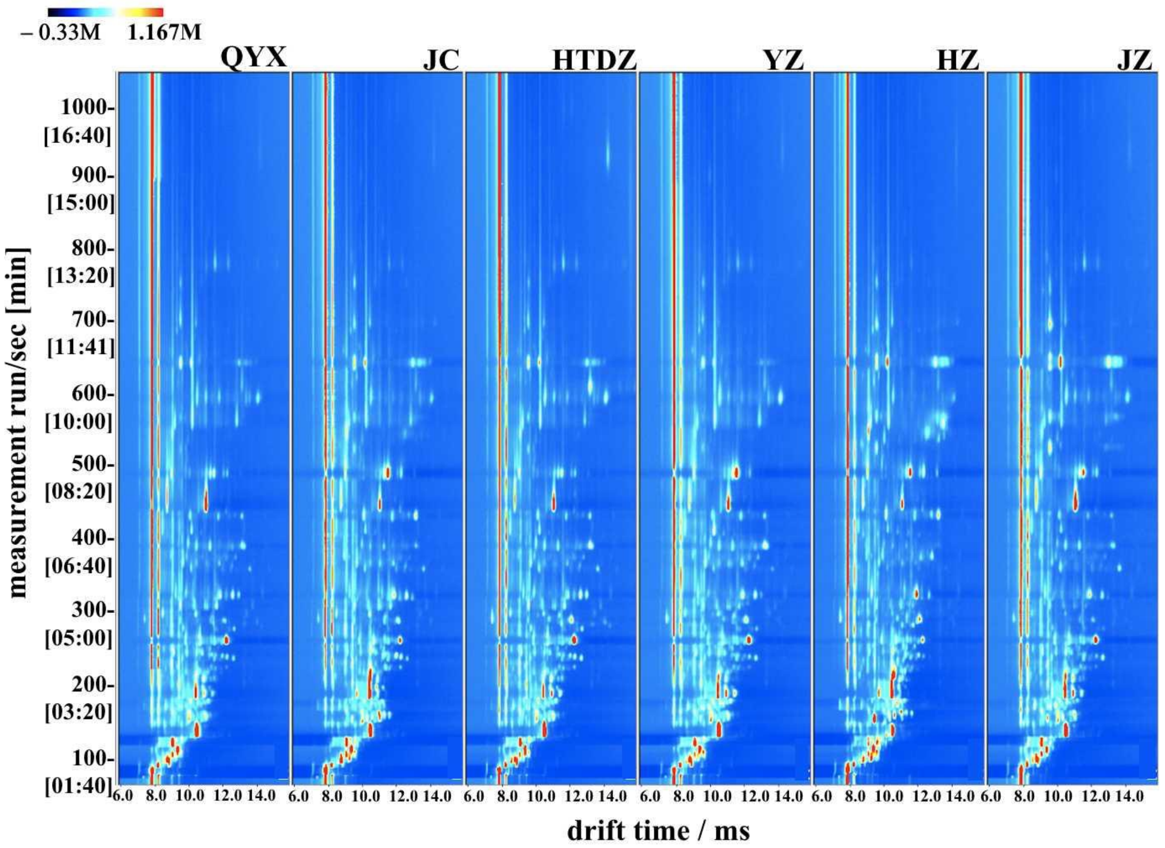


**Figure S1** The combined chromatograms of the six red jujube cultivars in Xinjiang province, China (Jinchang-'JC', Junzao-'JZ', Huizao-'HZ',Qiyuexian-'QYX', Hetiandazao-'HTDZ', and Yuanzao-'YZ')

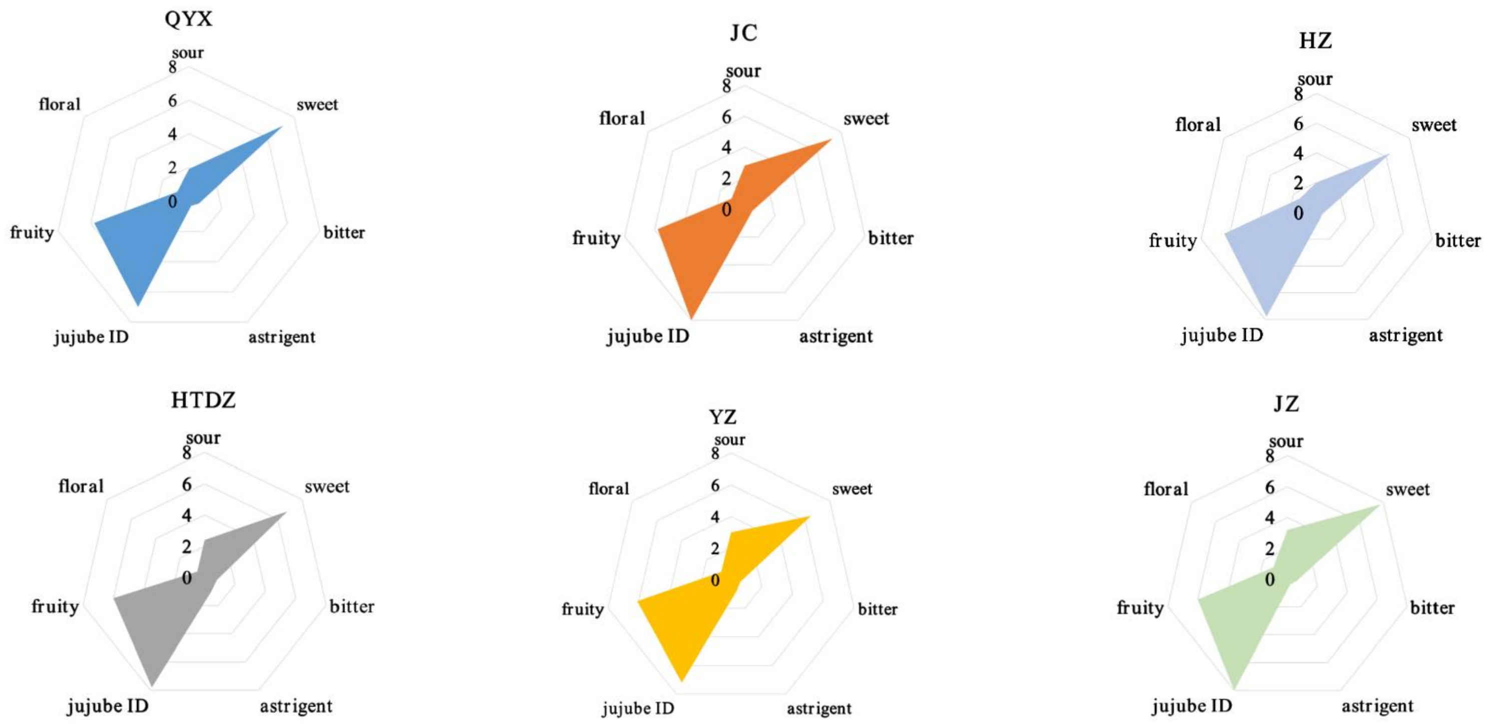


**Figure S2** Sensory profiles of the six red jujube cultivars in Xinjiang province, China (Jinchang-'JC', 'blue color'; Junzao-'JZ', 'orange color' , Huizao-'HZ', 'light blue color'; Qiyuexian-'QYX', 'grey color'; Hetiandazao-'HTDZ', 'light orange color'; and Yuanzao-'YZ', 'green color')

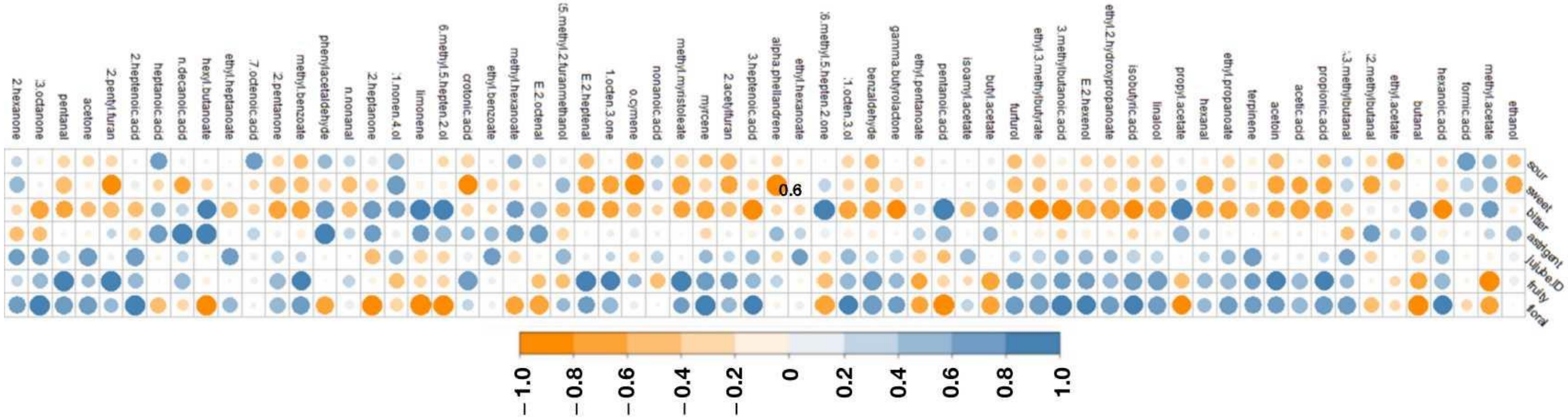


**Figure. S3** Correlation analysis among sensory attributes and VOCs of the six red jujube cultivars from Xinjiang province, China (Jinchang-'JC', Junzao-'JZ', Huizao-'HZ',Qiyuexian-'QYX', Hetiandazao-'HTDZ', and Yuanzao-'YZ')
